# 3D-printing process design of lattice compressor impeller based on residual stress and deformation

**DOI:** 10.1038/s41598-019-57131-1

**Published:** 2020-01-17

**Authors:** Dejun JIA, Fanchun LI, Yuan ZHANG

**Affiliations:** School of Ship and Ocean Engineering, Dalian Maritime University, Dalian, China

**Keywords:** Mechanical engineering, Aerospace engineering

## Abstract

The application of a lattice structure in the lightweight design of compressor impellers can reduce their mass and moment of inertia, hence improving the effective carrying of aircraft and reducing the start and braking moments of the impeller. The feasibility of a processing-lattice compressor impeller is the prerequisite for its application. To control the residual deformation and residual stress effectively, a computer-aided design technique is used to simulate the manufacturing process of a compressor impeller. The residual deformation and stress of the compressor impeller during the additive manufacturing process is calculated. The material-stacking process and base-plate- and support-removal process of a TiAl6V4 impeller printed by an SLM280 metal 3D printer are simulated by the finite-element method. The results show that some change in the laser printing parameters leads to a significant impact on the residual stress and deformation amplitude of the impeller. The residual deformation and residual stress of the lattice compressor impeller with the same geometrical appearance after processing are less than the corresponding amplitude of the solid compressor impeller, which also shows that the printed lattice compressor impeller can more easily achieve the design requirements.

## Introduction

Computer-aided design (CAD) technology has been widely used in the aerospace field, including for the design of traditional compressor impellers^1,2^. However, with the progress of science and technology and the continuous increase in industry demand, traditional product design and CAD technology can no longer meet these needs. For instance, reducing the structural mass of aircraft can improve the effective carrying of aircraft, increase the traversed distance of aircraft, and in some cases, improve the performance of the aircraft^3–5^. The application of a lattice structure can realize a lightweight aircraft structure design. The application of lattice structure in the lightweight design of the compressor impeller structure can not only reduce the overall structural mass of the aircraft, but also reduce the starting torque of the impeller. In this case, the interaction moment between the compressor impeller and the wheel axle is also reduced, the wheel axle structure can also be lightweight, and the structural mass of the aircraft is further reduced. Therefore, to ensure the structural safety and aerodynamic performance, a metal lattice impeller has great prospects for application in the aviation field. However, the processing of a lattice structure cannot be realized by traditional technology. The production of the lattice compressor impeller is a precondition of studying the structure and aerodynamic performance of the impeller, in addition to the mass production of the impeller. With the continuous progress in industrial manufacturing technology in recent years, additive manufacturing technology has attracted much attention because of its flexible design, strong machinability of complex structures, and shorter production cycle than traditional manufacturing technology. As a new technology, 3D-printing technology is gradually being accepted by engineers, and widely used in medical^6^, aerospace^7–10^, and other fields. With the development of 3D-printing technology and CAD technology, engineers have applied CAD technology widely in the design of lightweight lattice structures and the industrial design of additive manufacturing^11–14^. Accordingly, engineers can design more mechanisms that cannot be processed by traditional manufacturing technology that not only meet the requirements of the work, but also provide superior performance. Such mechanisms include aerospace structures based on topology optimization^7,8^ and functional gradient lattice structures based on topology optimization^15–17^. This type of structure can greatly reduce the mass of the structure, and adjust the optimization direction according to the designer’s needs so that the structure can have reduced mass and meet the needs of the specific environment. For example, Albert To^15^ used the progressive homogenization method to design a functional gradient lattice structure with elastic properties equivalent to those of a solid structure, based on the results of topological optimization. This resulted in higher first-order natural frequencies than the original structure. The application of a lattice structure in the lightweight design of parts can also reduce the vibration and noise of parts, while also reducing the structural mass. This is conducive to the absorption of collision energy^18^ and provides a safer and more comfortable environment for customers. The production of a metal lattice impeller can also be realized by metal 3D-printing technology.

Although metal 3D-printing technology can realize the production of a lattice compressor impeller, there are still many problems affecting the processing accuracy, and the rate of qualified products in the actual metal 3D-printing operation. Generally, the melting point of metal powder is higher than that of the metal powder melting bed in the process of 3D printing. This environment can lead to rapid cooling of the processed parts. The metal powder is heated continuously by laser and then cooled in cooling gas after melting. The temperature difference between the time and space caused by this process leads to a series of permanent non-design deformations and high residual stress^19,20^. Therefore, to meet the accuracy requirements of the processed parts, the direct application of metal 3D-printing technology to the processing of the processed parts usually requires repeated processing of the same parts to ensure that the accuracy of the processed parts gradually meet the accuracy requirements^21,22^. This repeated process is cumbersome and leads to higher production costs. In addition, the cooling process of metal 3D printing is different from that of traditional forging and casting processes. Different cooling processes lead to different crystalline structures, and the difference in microstructure directly influences the mechanical properties (such as modulus and yield strength) of the processed parts^23^. To predict the mechanical properties (such as deformation and residual stress), shorten the design and production cycle, reduce the production cost of the processed parts, and improve the qualified product rate, it is necessary to apply CAD technology to simulate the production process of metal 3D printing when adding materials to the processed parts (including the metal lattice impeller). Currently, some scholars have carried out a number of experimental studies on the mechanical properties^15,18,24–26^ and 3D-printing process^27–30^ of lattice structures. In these studies, some simple geometric structures are taken as the main research objects, and the mechanical properties of these lattice structures and effects of the printing parameters on the mechanical properties of these structures are analyzed. The thermal history of simulated additive manufacturing process is similar to that of simulated multi pass welding^31–33^. In the aspect of 3D printing heat transfer simulation, the finite element modeling technology of metal deposition heat transfer analysis in additive manufacturing process was studied in detail^34^. Besides, a new method of hybrid quiet non active metal deposition is proposed to accelerate the operation of the computer. The effects of different laser scanning paths and different units on printing results were simulated by numerical simulation^35,36^. Although these studies are aimed at simple models, they are of great significance. In addition, there is no research on the performance and processing feasibility of the lattice compressor impeller. However, ensuring the feasibility of processing the lattice compressor impeller is the prerequisite for its application. A study on the printing process simulation of compressor impeller can explore the feasibility of 3D printing process application in related fields.

For these reasons, based on the CAD technique and the finite-element method (FEM), the manufacturing process of the compressor impeller is simulated, and the residual deformation and residual stress of the compressor impeller during the printing process are calculated. The material-stacking process and base-plate- and support-removal process of the impeller are simulated by FEM, and the predicted deformation of the impeller without heat treatment is obtained. In addition, the influence of different printing parameters on the maximum residual stress and total displacement at different sections of impeller structure are studied by changing the printing parameters. The TiAl6V4 impeller is printed with a SLM280 Metal 3D Printer, a 3D scanner is used to scan the printed impeller, and the numerical geometry model of solid impeller is established based on the obtained point-cloud data. The printed impeller geometry model obtained by numerical calculation, solid impeller numerical geometry model, and original design geometry model, are compared. Based on deformation deviation, the feasibility evaluation of the metal printing process simulation based on FEM is carried out. This research provides a basis and reference for further turbo production and performance design. Furthermore, the residual stress and deformation of solid and lattice impellers in different working conditions are compared. Based on these results, the machinability of two different impellers is evaluated. Based on the above results, suggestions for impeller design and selection are presented.

## Result

### Compressor in this study

The solid compressor impeller selected in this paper is from a certain type of compressor. Figure S.1(a,b) show the impeller and its profile with a lightweight lattice, respectively. The mass of the impeller is reduced from 195.3 to 149.4 g. The lattice structure is applied to the lightweight design of the impeller, which decreases the mass of the impeller by 45.9 g. The operation condition of the impeller can be seen in Supplementary (Table S.1). The frequencies corresponding to the first three nodal-diameter modes of the impeller are also described in the Supplementary (Table S.2).

### Numerical simulation of production process for lattice compressor impeller

After the lightweight design of the lattice impeller is completed, the 3D-printing process of the impeller is simulated by a numerical method, and the effect of the printing parameters on the residual deformation and stress of the impeller is studied. An SLM280 laser powder melting bed metal 3D printer and TiAl6V4 alloy powder are selected for printing the impeller. Some of the main parameters in the printing process are shown in Table S.3. The powder properties are shown in Tables S.4, S.5 and S.6. Based on many calculation results, it is found that when the mesh size is 1 mm, the reduction in the mesh size has very little influence on the simulation results. Furthermore, a 1-mm mesh can ensure that the simulation time is acceptable. Therefore, the mesh size of the simulation experiment is set to 1 mm in this section. Figure S.2 shows the mesh of the impeller and supports. The results of the meshing impeller and support structures are shown in Fig. S.3. In this case, the whole printing part is divided into 53 layers in the vertical direction.

**Figure 1:**
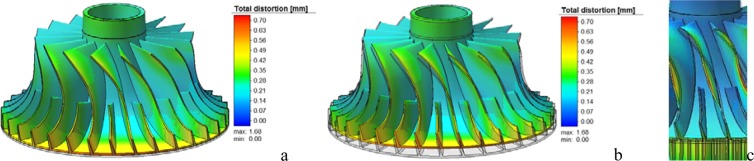
Deformation comparison between original model and simulation results|| (**a**) Primary proportional display; (**b**) Five-fold proportional display; (**c**) Blade deformation direction.

Figure 1(a) shows the deformation of the impeller relative to the original design. To observe the details of impeller deformation more clearly, the actual deformation is enlarged by 5 times. Figure 1(b) shows the deformation of the impeller after enlarging 5 times, in which the grey part is the design model without deformation. Comparing the design model with the deformed results, it is found that the deformed direction of the impeller blade surface is the direction of decreasing the curvature of the blade surface, as show in Fig. 1(c). In addition, because the local deformation is too large, the shroud side of the impeller shows a small irregular convex or concave deformation.

### Additive manufacture of the solid model of lattice compressor impeller and the verification of feasibility of numerical method

**Figure 2:**
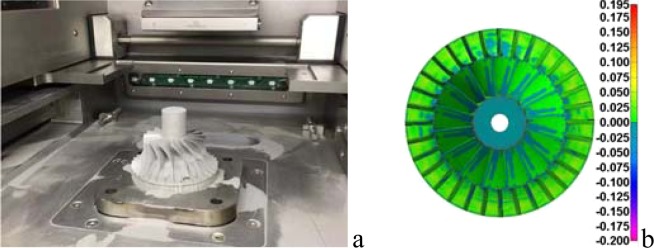
Printed impeller and comparison with simulation results|| (**a**) Printed impeller with support; (**b**) Printed impeller to compare with simulation results (mm).

To verify the accuracy of the simulation results and the practicability of the simulation method, a solid model of the lattice structure compressor impeller is printed with a 3D printer in this section, and the print results are compared with the design model. In this paper, SLM280 selective laser powder melt metal 3D printer is used for compressor impeller printing. The main parameters of the printing process are set in Table 1. The laser starting angle is 15°, and the laser deflection is 67° after each layer is printed. The impeller after preliminary printing is shown in Fig. 2(a). After the support is removed, milling is applied to further treat the bottom of the hub to reduce the roughness of the bottom of the hub. Here, the roughness of the printed impeller is 6.3, and that of the milled impeller is 1.6. The simulation geometry file is compared with the geometry file obtained by scanning the solid (Fig. S.6). The results show that the numerical method can simulate the printing process better (Figs. 2(b) and S.7). Refer to “method” for scanning method, See supplementary for scanning path of the laser during the printing progress and solid impeller.

**Table 1:** Relative deviation of residual deformation and residual stress of lattice and solid compressor impellers.

Work case	Relative deviation of residual deformation	Relative deviation of residual stress
1	4.91%	6.87%
2	20.19%	0.61%
3	2.73%	6.91%
4	14.83%	8.47%
5	3.32%	8.72%

### Residual deformation and stress of compressor impeller at different conditions

Here, the other parameters of Table S.3 remain unchanged, while the laser power of the printer is changed. In this case, the base plate and support are not yet removed. The maximum residual deformation and stress of the printed piece during the printing process are in supplementary. Here, the number of layers is the number of grid layers, not the number of powder layers.

**Figure 3:**
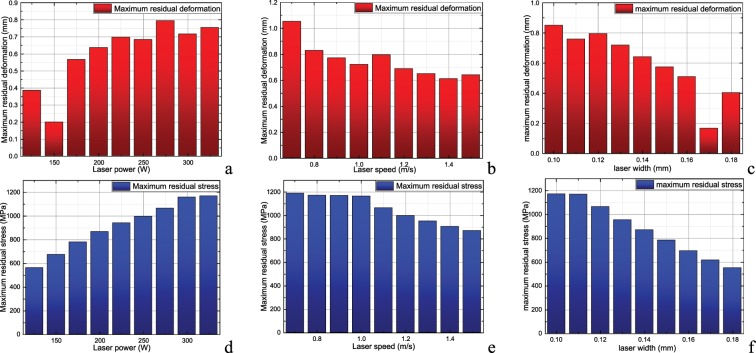
Maximum residual deformation and maximum residual stress of impeller (after removal of base plate and support)|| (**a**) Maximum residual deformation at different laser power; (**b**) Maximum residual deformation at different laser speed; (**c**)Maximum residual deformation at different laser width; (**d**) Maximum residual stress at different laser power; (**e**) Maximum residual stress at different laser speed; (**f**) Maximum residual stress at different laser width.

Figure 3(a,d) shows the maximum residual deformation and residual stress of the printed piece at different laser power. Here, the impeller’s base plate and support structure have been removed. As shown in Fig. 3(d), when the laser power increases, the final residual stress amplitude increases after the impeller is printed, which is due to the higher processing temperature difference caused by the high laser power. In this case, due to the removal of the base plate and the support structure, some stress of the impeller is released, so the stress amplitude of the impeller is lower than that before the removal of the base plate and the support. Figure 3(b,e) shows the maximum residual deformation and residual stress of the printed piece at different laser speeds. Here, the impeller’s base plate and support structure have been removed. As shown in Fig. 3(e), when the laser power increases, the final residual stress amplitude of the impeller after printing decreases accordingly. This is because of the lower local heating time caused by the high laser speed, resulting in a lower processing temperature difference. In addition, the removal of the base plate and the support reduces the residual stress of the impeller, and the selection of different reference points reduces the observed value of residual deformation of the impeller. Figure 3(c,f) shows the maximum residual deformation and residual stress of the printed piece at different laser widths. Here, the impeller’s base plate and support structure have been removed. Figures S.10(a) and 3(c), it can be seen that the difference of reference points leads to the increase in maximum deformation observation value after the removal of base plate and support. From Figs. S.10(b) and 3(f), it can be seen that the residual stress of impeller is released after the base plate and support are removed. Moreover, increasing the laser width causes the printed parts to heat more uniformly, and the maximum stress decreases with the increase in laser width.

**Figure 4:**
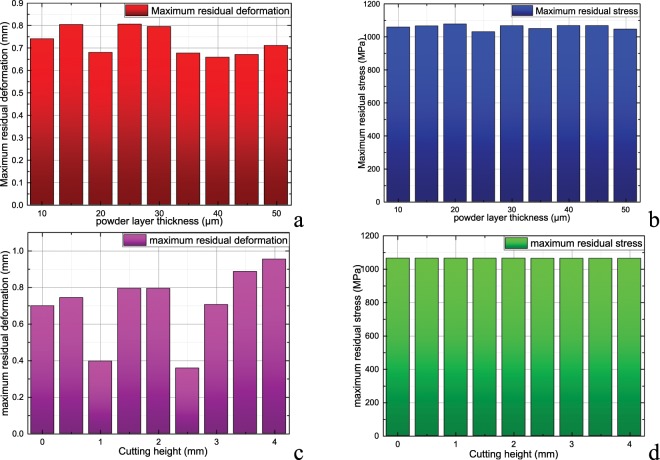
Maximum residual deformation and maximum residual stress of impeller (after removal of base plate and support)|| (**a**) Maximum residual deformation at different powder layer thickness; (**b**) Maximum residual stress at different powder layer thickness; (**c**) Maximum residual deformation at different cutting height; (**d**) Maximum residual stress at different powder layer thickness.

Figure 4(a,b) shows the maximum residual deformation and residual stress of the printed piece at different powder layer thicknesses. Here, the impeller’s base plate and support structure have been removed. The influence of changing the thickness of the powder layer on the residual deformation and residual stress amplitude of impeller after removal of support is obviously weaker than that of changing the laser parameters. Figure 4(c,d) shows the maximum residual deformation and residual stress of impeller after removal of the support and base plate at different cutting heights. From Fig. 4(c,d), it can be seen that the cutting height of the base plate and the support after printing influences the residual deformation of the impeller, and the effect on the residual strain is between 0.01 and 0.1 MPa. A reasonable base plate cutting height determines the final impeller shape, but does not influence the ultimate safety of the impeller structure. Therefore, when choosing the cutting height, the final shape of the impeller can be taken as the goal without considering the structural safety of the impeller.

### Comparison of printing results between solid compressor impeller and lattice compressor impeller

**Figure 5:**
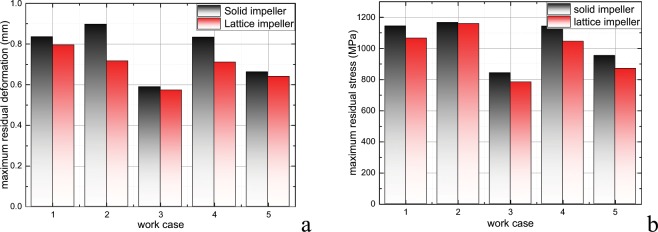
Maximum residual deformation and maximum residual stress of impeller at different work case (after removal of base plate and support)|| (**a**) Maximum residual deformation at different work case; (**b**) Maximum residual stress at different work case.

At present, research on the manufacturing technology of solid compressor impellers is not yet perfect, but some progress has been made. Its processing difficulty is mainly in terms of controlling deformation and guaranteeing consistency. The residual deformation and stress of the lattice and solid compressor impellers after finishing the printing operation and removing the base plate and support at different working conditions are calculated in this section. By comparing the residual deformations and stresses, the machinability of lattice compressor impellers is studied. Based on the comparison results, some suggestions for the design of printing operation are provided. Figure 5 shows the maximum residual deformation and residual stress of printed parts after removal of the base plate and support at different printing conditions. Table 1 shows the relative deviation in the residual deformation and residual stress of the lattice and solid compressor impellers. Figures S.12 and S.13 shows the residual deformation and stress distribution of the solid and lattice impellers after removal of the base plate and support in printing work case 1, respectively. By comparing the residual stress and deformation amplitude, and the distribution of the solid and lattice compressor impellers at different printing conditions, it is found that the residual stress and deformation of the lattice impeller are less than those of the solid impeller under the same printing parameters. In the current research, the residual deformation of the lattice compressor impeller can be reduced by 20.19% compared with that of the solid compressor impeller. This means that the original design of the lattice impeller is easier to approach after processing than the solid impeller. Therefore, the aerodynamic performance of the lattice impeller is closer to the original design then that of the solid one. In addition, the residual stress of the lattice impeller is smaller than that of solid impeller. In the case of the current research, the residual stress of the lattice compressor impeller can be reduced by 8.72% compared with that of solid compressor impeller. This means that the lattice impeller is less vulnerable to damage than the solid impeller after finishing the impeller processing. One of the possible reasons for this phenomenon is that the lattice impeller structure can more easily radiate heat during the printing process. This conjecture can be verified by further research, and other possible causes of the above phenomena can be explored. Furthermore, the lattice compressor impeller is more practical than the solid compressor impeller if it can ensure the structural safety of the impeller in operation. The structural safety and stability of a lattice compressor impeller can be studied by subsequent research.

In addition, compared with the design, the maximum deviation of solid impeller processed by milling method is 0.19 mm. Its accuracy is obviously higher than that of solid impeller made of additive. For the manufacturing of lattice compressor impeller, the combination of 3-D printing and milling can also be considered.

## Discussion

In this paper, an octagonal truss lattice structure is applied to the lightweight design of a compressor impeller. The mass of the impeller decreases from 195.3 g to 149.4 g after replacing the solid area inside the hub with the lattice structure. Subsequently, the residual deformation and stress of lattice compressor impeller during printing process at different working conditions are calculated based on FEM and CAD techniques. Furthermore, the residual deformation and maximum residual stress of the lattice impeller after removal of the base plate and support are calculated. The feasibility of the computer-aided technique to simulate the printing process is preliminarily verified by comparing the calculated results with the geometric shape of the printing results. Finally, the residual stresses and deformations of solid compressor impeller and lattice compressor impeller after finishing printing operation and removing the support and base plate at different working conditions are calculated. The following conclusions are reached based on the results:

1. The simulation results show that the deformation distribution of the impeller has the same trend in space as that of the final solid model of impeller, and there are differences in the amplitude. The mutual occlusion and operation errors of the models partially distort the scanning model. Generally, the simulation results are in good agreement with the real printing results.
2. At different printing parameters, the trends of the deformation and maximum stress of the impeller in the printing process are similar to each other (see also Figs. S.8 to 11), but the amplitudes are different. Compared with the thickness of the powder coating, laser parameters (power, speed, and width) have a greater impact on the final printing results. The residual stress of the impeller can be reduced by decreasing the laser power, increasing laser speed, and increasing the laser width. For example, when the laser power is less than 275W, the laser speed is more than 1 m/s, and the laser width is more than 0.12 mm, the maximum stress of the lattice impeller is within the safe range. In these cases, the residual deformation of impeller will also decrease with increasing the laser power, increasing laser speed, and increasing the laser width. Further, removal of the support and base plate can release part of the residual stress. In addition, it should be noted that the metal powder needs to be melted in the process of 3D printing. When controlling the residual stress and deformation of impeller with the above method, it is necessary to consider whether the heating temperature can reach the powder melting point and whether the heating time can ensure that the metal powder melts by absorbing enough heat.
3. In this research, the residual deformation of lattice compressor impeller can be reduced by 20.19% compared with that of the solid compressor impeller. Compared with a solid impeller, the lattice impeller can more easily achieve the original design after processing. In addition, the residual stress of the lattice impeller is smaller than that of solid impeller, and the maximum residual stress can be reduced by 8.72%.

In this paper, a new lightweight-structure with application prospects is designed, and its manufacturing process is simulated by numerical methods. The feasibility of the process is proven by comparison with the actual product and design. In this paper, the rotor dynamics, structural safety, and reliability of the new have not been analyzed. The mechanical properties of the lattice can be evaluated by further research. In addition, in view of the deformation of the, the printing compensation design of the can be further studied.

Besides, a weak coupling relationship between thermal analysis and mechanical analysis is assumed in the calculation of thermal and mechanical problems in 3D metal printing. However, in the actual printing process, due to the thermal expansion of the metal, its outer boundary will change. The boundary conditions in the next iteration will change accordingly. The input heat will change, which will cause the difference of the structure temperature change, and then affect the deformation of the structure. For small deformation problems, the influence is relatively small. However, such weak coupling is not accurate for structures with large size or large deformation, a complete thermo mechanical coupling model should be established to simulate large-scale structure or large deformation structure.

In the process of verifying the machinability of 3-D printing wheel, the feasibility of numerical method in simulating deformation is verified by using fitting method. However, it is also very important to measure the corresponding stress. The measurement of impeller stress can be carried out in the future research.

We mainly focus on the machinability of impeller in this study. However, the rotor dynamic characteristics of impeller are also very important. This study has proved that lattice impeller has excellent machinability, Therefore, in the future research, we can take the rotor dynamic characteristics of the impeller as the goal to design a lattice compressor impeller that meets the specific requirements.

## Method

### Basic theory of thermal and mechanical computation for metal additive manufacturing

In this section, the thermal and mechanical behavior in the process of additive manufacturing, as well as the numerical calculation equation and calculation method describing these behaviors, are introduced. First, the Galerkin method is applied to transform the physical governing equation into a weak form. The energy-balance equation is the governing equation of the thermal problem, and the stress balance equation is the governing equation of the mechanical problem. The nodal displacement solution vectors U, residual vectors R, and stiffness matrix dR/d*T* of the process can be derived from the weak-form control equation. Starting from the preliminary estimation of U^0^, the Newton–Raphson method is applied to the iterative process in the form of:

1 $${U}^{i+1}={U}^{i}-{[\frac{{\rm{d}}{R}^{i}}{{\rm{d}}U}]}^{-1}{R}^{i}$$

Here, *i* and *i* + 1 represent the ordinal number of the previous iteration step and the current iteration step^37^, respectively. In each time step, the solution of the previous time step is used as the initial value of the current time step. In the analysis of engineering thermodynamic problems, the finite element formulas for quasi-static thermo-elastic-plastic processes in a Lagrangian reference frame have been widely used^38–40^. This method assumes that thermal analysis is transient, while elastic-plastic analysis is quasi-static. In many thermo-elastic-plastic process analyses, such as welding and thermo-assisted forming, it is usually assumed that there is a weak coupling between mechanical and thermal variables, meaning temperature distribution is independent of stress and strain. Therefore, in the numerical calculation of simulating the 3D-printing process, heat transfer analysis is carried out first, and the temperature distribution is regarded as the import load of mechanical analysis. Thermal and mechanical problems can be considered as non-linear problems based on material temperature characteristics and mechanical properties^34^.

### Transient thermal analysis

In the Lagrangian reference system, the thermal balance equation in volume domain *V* has the following forms^34^:

2 $${\rm{Q}}({\boldsymbol{X}},t)-\frac{dH}{dt}({\boldsymbol{X}},t)-\nabla \cdot {\bf{q}}({\boldsymbol{X}},t)=0$$

Here, ***X*** is the spatial coordinate, t is time, **q** is the heat-flux vector, Q is the volume heat source, and *H* is enthalpy.

The initial temperature of volume domain *V* is given by the following equation:

3 $$T({\boldsymbol{X}},{t}_{0})={T}_{0}({\boldsymbol{X}})$$

Here, T is the temperature and *T*_0_(***X***) is the initial temperature. The boundary conditions of the equation are as follows:

4 $$T({\boldsymbol{X}},t)={T}_{p}({\boldsymbol{X}},t)$$

5 $${q}_{s}({\boldsymbol{X}},t)={q}_{p}({\boldsymbol{X}},t)$$

Here, and *T*_*p*_(***X***, *t*), *q*_*p*_(***X***, *t*), represent the specified temperature and temperature-related surface heat flux, respectively. The form of surface heat flux *q*_*p*_(***X***, *t*) corresponding to the surface convection heat transfer and surface radiation heat transfer is as follows:

6 $${q}_{p}({\boldsymbol{X}},t)=h(T-{T}_{\infty })+\varepsilon \sigma ({T}^{4}-{T}_{\infty }^{4})$$

Here, *h* is the convection coefficient, *T*_∞_ is the ambient temperature, *ε* is emissivity, and σ is the Boltzmann constant. The energy flux *q* is expressed as a function of temperature T by the constitutive relation of nonlinear isotropic Fourier heat flux:

7 $${\bf{q}}=-\,k(T)\nabla T$$

Here, *k* is thermal conductivity. The form of enthalpy variability is

8 $$\frac{{\rm{d}}H}{{\rm{d}}t}=\frac{{\rm{d}}H}{{\rm{d}}T}\frac{{\rm{d}}T}{{\rm{d}}t}=\rho {C}_{p}\frac{{\rm{d}}T}{{\rm{d}}t}$$

Here, *ρ* is fluid density, and *C*_*p*_ is the specific heat. From Eqs. (2), (7), and (8), we have

9 $${\rm{Q}}({\boldsymbol{X}},t)-\rho {C}_{p}\frac{dT}{dt}\nabla \cdot [k(T)\nabla T]=0$$

By using the implicit finite-difference method, the relationship between temperature and time is as follows:

10 $$\frac{{\rm{d}}{T}_{n}}{{\rm{d}}{t}_{n}}\cong \frac{{T}_{n}-{T}_{n-1}}{{t}_{n}-{t}_{n-1}}$$

Here, *T*_*n*_ and *T*_*n−*1_ are the temperatures at time *t*_*n*_ and *t*_*n−*1_, respectively. The residual vectors R and Jacobian term d***R***/d**T**_*n*_ can be obtained by the Galerkin finite-element discretization method, and the Newton–Raphson solution format after using Eqs. (9) and (5) is

11 $${\bf{R}}={\int }_{{V}_{element}}\{{{\bf{B}}}^{T}{\bf{kB}}{{\bf{T}}}_{n}-{{\bf{N}}}^{T}Q+{{\bf{N}}}^{T}{\bf{N}}\rho {C}_{p}\frac{{{\bf{T}}}_{n}-{{\bf{T}}}_{n-1}}{{t}_{n}-{t}_{n-1}}\}{\rm{d}}V+{\int }_{{A}_{qelement}}{{\bf{N}}}^{T}{q}^{p}dA$$

12 $$\begin{array}{rcl}\frac{{\rm{d}}{\bf{R}}}{{\rm{d}}{{\bf{T}}}_{n}} & = & {{\int }^{}}_{{V}_{element}}[{{\bf{B}}}^{{\boldsymbol{T}}}{\bf{kB}}+{{\bf{B}}}^{{\boldsymbol{T}}}\frac{\partial {\bf{K}}}{\partial {\boldsymbol{T}}}{{\bf{B}}}^{{\boldsymbol{n}}}{\bf{TN}}-{{\bf{N}}}^{{\boldsymbol{T}}}\frac{\partial {\boldsymbol{Q}}}{\partial {\boldsymbol{T}}}{\bf{N}}+{{\bf{N}}}^{{\boldsymbol{T}}}{\bf{N}}{\boldsymbol{\rho }}{{\boldsymbol{C}}}_{{\boldsymbol{p}}}\frac{1}{{t}_{n}-{t}_{n-1}}]{\rm{d}}{\boldsymbol{V}}\\  &  & +{\int }_{{V}_{element}}[{{\bf{N}}}^{T}{\bf{N}}\rho \frac{\partial {C}_{p}}{\partial T}{\bf{N}}\frac{{{\bf{T}}}_{n}-{{\bf{T}}}_{n-1}}{{t}_{n}-{t}_{n-1}}]{\rm{d}}V+{\int }_{{A}_{qelement}}{{\bf{N}}}^{T}\frac{\partial q}{\partial T}{\bf{N}}{\rm{d}}A\end{array}$$

Here, **T** is the nodal temperature vector, and **N** and **B** are the temperature and temperature gradient operators, respectively. Then, we have

13 $$T={\bf{NT}}$$

14 $$\nabla T={\bf{BT}}$$

To solve these equations it is necessary to have an initial condition, a heat input model, and thermal boundary conditions. The initial condition is set to the temperature of either the ambient or preheating temperature for the substrate or build plate elements. The heat input model may be an applied heat flux or volumetric heat source model. The boundary conditions are usually set as ambient temperature and substrate temperature. In this study, the substrate temperature was 200 °C, and the ambient temperature was about 45 °C.

### Mechanics analysis

The material equilibrium equation with volume V and boundary A can be written as follows^41^:

15 $$\nabla \cdot {\bf{S}}+{\bf{b}}=0$$

Here, **S** is the second-order stress tensor and **b** is the volume-force vector. The boundary conditions of the same material are.

16 $${\bf{u}}=\bar{{\bf{u}}}$$

17 $${\bf{Sn}}=\bar{{\bf{t}}}$$

Based on the small-deformation theory, the relationship between strain tensor **E** and displacement vector **u** is as follows:

18 $${\bf{E}}=\frac{1}{2}\{\nabla {\bf{u}}+{[\nabla {\bf{u}}]}^{{\rm{T}}}\}$$

Because of symmetry, stress tensor **S** and strain tensor **E** can be expressed in vector form as **σ** and **ε**. According to the hypothesis of small deformation and thermo-elastic-plastic theory, the total strain can be decomposed into three parts: elastic strain **ε**, plastic strain **ε**_*p*_, and thermal strain **ε**_*t*_. The relationship is

19 $${\boldsymbol{\varepsilon }}={{\boldsymbol{\varepsilon }}}_{e}+{{\boldsymbol{\varepsilon }}}_{p}+{{\boldsymbol{\varepsilon }}}_{t}$$

The initial conditions can be expressed as

20 $$\begin{array}{ccc}{\bf{u}} & = & {{\bf{u}}}^{0}\\ {{\boldsymbol{\varepsilon }}}_{p} & = & {{\boldsymbol{\varepsilon }}}_{p}^{0}\\ {{\boldsymbol{\varepsilon }}}_{q} & = & {{\boldsymbol{\varepsilon }}}_{q}^{0}\end{array}$$

Here, **ε**_*q*_ is the equivalent plastic strain. The stress–strain relationship is

21 $${\boldsymbol{\sigma }}={\bf{C}}{{\boldsymbol{\varepsilon }}}_{e}={\boldsymbol{\varepsilon }}-{{\boldsymbol{\varepsilon }}}_{p}-{{\boldsymbol{\varepsilon }}}_{t}$$

Here, **C** is the elastic tensor related to the temperature. Similar to the thermal analysis equation, the element residual **R** can be obtained by using the weak form of the equation and the finite-element discretization method:

22 $${\bf{R}}({{\bf{U}}}_{n})=\sum _{G{V}_{e}}\,[{{\bf{B}}}^{T}{{\boldsymbol{\sigma }}}_{n}-{{\bf{N}}}^{T}{\bf{b}}]WJ+\sum _{G{A}_{e}^{t}}\,{{\bf{N}}}^{T}\bar{{\bf{t}}}wj$$

here, **U** is element displacement vector. The stress related to time t obtained by the discretization method can be described as follows:

23 $${{\boldsymbol{\sigma }}}_{n}={{\boldsymbol{\sigma }}}_{n-1}+\Delta {\boldsymbol{\sigma }}$$

here, Δ denotes the increment between *t*_*n*−1_ and *t*_*n*_. Equation (19) is solved at *t*_*n*−1_ and *t*_*n*_, and the difference is obtained as follows:

24 $$\Delta {\boldsymbol{\sigma }}={{\bf{C}}}_{n}[\Delta {\boldsymbol{\varepsilon }}-\Delta {{\boldsymbol{\varepsilon }}}_{p}-\Delta {{\boldsymbol{\varepsilon }}}_{t}]+\Delta {{\bf{C}}}^{{\boldsymbol{n}}-1}{{\boldsymbol{\varepsilon }}}_{e}$$

Here:

25 $$\Delta {\boldsymbol{\varepsilon }}={\bf{B}}[{{\bf{U}}}_{n}-{{\bf{U}}}_{n-1}]$$

26 $$\Delta {{\boldsymbol{\varepsilon }}}_{p}=\Delta {{\boldsymbol{\varepsilon }}}_{q}{\bf{a}}$$

27 $$\Delta {{\boldsymbol{\varepsilon }}}_{t}=[{\varepsilon }_{{t}_{n}}-{\varepsilon }_{{t}_{n-1}}]{\bf{j}}=\{{\alpha }_{n}[{T}_{n}-{T}^{{\rm{ref}}}]-{\alpha }_{n-1}[{T}_{n-1}-{T}^{{\rm{ref}}}]\}{\bf{j}}$$

28 $${\bf{j}}={[\begin{array}{ccc}\begin{array}{ccc}\begin{array}{cc}1 & 1\end{array} & 1 & 0\end{array} & 0 & 0\end{array}]}^{{\rm{T}}}$$

where **a** is the stream vector, α is the coefficient of thermal expansion r *T*^ref^ is the reference temperature. The predicted values of linear elastic stress ***σ***_*B*_ and corresponding elastic strain ***ε***_*Be*_ are defined as follows:

29 $${{\boldsymbol{\sigma }}}_{B}={{\boldsymbol{\sigma }}}_{n-1}+{{\bf{C}}}_{{\boldsymbol{n}}}[\Delta {\boldsymbol{\varepsilon }}-\Delta {{\boldsymbol{\varepsilon }}}_{{\rm{t}}}]+\Delta {{\bf{C}}}^{n-1}{{\boldsymbol{\varepsilon }}}_{{\rm{e}}}$$

30 $${{\boldsymbol{\varepsilon }}}_{Be}={{\boldsymbol{\varepsilon }}}_{{e}_{n-1}}+\Delta {\boldsymbol{\varepsilon }}-\Delta {{\boldsymbol{\varepsilon }}}_{{\rm{t}}}$$

The yield function can be given by the following formula by using corresponding J2 elastic-plastic model^42^:

31 $$f={{\boldsymbol{\sigma }}}_{m}-{{\boldsymbol{\sigma }}}_{{\rm{y}}}$$

here, *σ*_*m*_ and *σ*_Y_ are the Mises stress and yield stress, respectively. Active yielding of structures occurs when *f* ≥ 0; *σ*_*B*_ and ***ε***_*Be*_ can be simplified to ***σ****n* and ${{\boldsymbol{\varepsilon }}}_{{e}_{n}}$, respectively, when the structure yields actively; and Δ**ε**_q_ can be calculated by the radial round-trip algorithm^39^. The element stiffness matrix is given by the following formula:

32 $$\frac{{\rm{d}}{\bf{R}}}{{\rm{d}}{{\bf{U}}}_{n}}=\sum _{G{V}_{e}}[{{\bf{B}}}^{{\rm{T}}}\frac{{\rm{d}}{{\boldsymbol{\sigma }}}_{n}}{{\rm{d}}{{\boldsymbol{\varepsilon }}}_{n}}{\bf{B}}-{{\bf{N}}}^{{\rm{T}}}\frac{{\rm{d}}{\bf{b}}}{{\rm{d}}{{\bf{U}}}_{n}}]WJ-\sum _{G{A}_{e}^{t}}{{\bf{N}}}^{{\rm{T}}}\frac{{\rm{d}}\bar{{\bf{t}}}}{{\rm{d}}{{\bf{U}}}_{n}}wj$$

$\frac{{\rm{d}}{{\boldsymbol{\sigma }}}_{n}}{{\rm{d}}{{\boldsymbol{\varepsilon }}}_{n}}$ is equal to **C**_*n*_ when non-active yielding of the structure occurs, and we have

33 $$\frac{{\rm{d}}{{\boldsymbol{\sigma }}}_{n}}{{\rm{d}}{{\boldsymbol{\varepsilon }}}_{n}}={\lambda }_{{\rm{eff}}}{\bf{j}}{{\bf{j}}}^{{\rm{T}}}+2{G}_{{\rm{eff}}}{{\bf{L}}}^{-1}+[\frac{3GH}{3G+H}-3{G}_{{\rm{eff}}}]{\bf{p}}{{\bf{p}}}^{{\rm{T}}}$$

in which

34 $${\bf{p}}=\frac{2}{3}{{\bf{L}}}^{-1}{\bf{a}}$$

35 $${G}_{{\rm{eff}}}=G\frac{{\sigma }_{{\rm{Y}}}}{{\sigma }_{Bm}}$$

36 $${\lambda }_{{\rm{eff}}}=\frac{3k-2{G}_{{\rm{eff}}}}{3}$$

37 $${\bf{L}}{\boldsymbol{=}}\text{diag}(1\,1\,1\,2\,2\,2)$$

Here, *G* is the shear modulus, *k* is the bulk modulus, *H* is the isotropic hardening coefficient, ***σ***_***B****m*_ is the Mises stress of the predicted value ***σ***_***B***_ of the linear elastic stress, and **p** is a conversion form of stream vector **a**.

### Surface fitting method

In this study, STL files are output to obtain the geometry files of simulation impeller and design impeller, and obtain the geometry file of the printing impeller through the scanner scanning technology. PolyWorks is applied to fit and compare the files.

**Figure 6:**
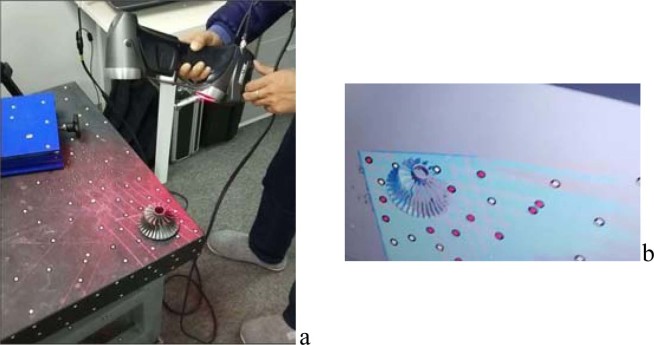
Scanning process of hand-held laser 3D scanner|| (**a**) 3D scanning operation; (**b**) Impeller display in scanning interface.

Because the lower bottom surface of the impeller is relatively flat and not easy to scan, a handheld laser 3D scanner is used in this section to scan the upper surface of the printed impeller. A handheld laser 3D scanner measures the spatial position of each laser reflection point (i.e., the set of points on the impeller surface) by measuring the time difference between the emitted and reflected laser beams. The scanned point-cloud data on the upper surface of the impeller is automatically stored in the computer. Figure 6 shows the process of scanning an impeller with a handheld laser 3D scanner and the display of the impeller surface in the scanning interface.

Because of the complex structure of the impeller surface, the large change in the surface curvature, and the mutual occlusion between blades and hub, part of the laser emitted by the 3D scanner returns to the scanner after more than one reflection on the impeller surface, which distorts some of the scanning results. Because of mutual occlusion, it is possible that part of the impeller surface cannot be scanned. In both cases, there may be a small amount of distortion in the scanning results, and there are some holes in the point-cloud geometry files obtained by scanning.

## Supplementary information

Supplementary Information.
