# Supplementary material for: 3D-printing process design of lattice compressor impeller based on residual stress and deformation
Source: Sci Rep. 2020 Jan 17;10:600. doi: 10.1038/s41598-019-57131-1 (PMC6969138; doi:10.1038/s41598-019-57131-1)
Supplement: Supplementary file 1 — Supplementary Information. [file 41598_2019_57131_MOESM1_ESM.doc]

Supplementary

3D-printing process design of lattice compressor impeller based on residual stress and deformation

Dejun JIA^1^, Fanchun LI^2^ , Yuan ZHANG^3^

^1,2,3^ School of Ship and Ocean Engineering, Dalian Maritime University

| 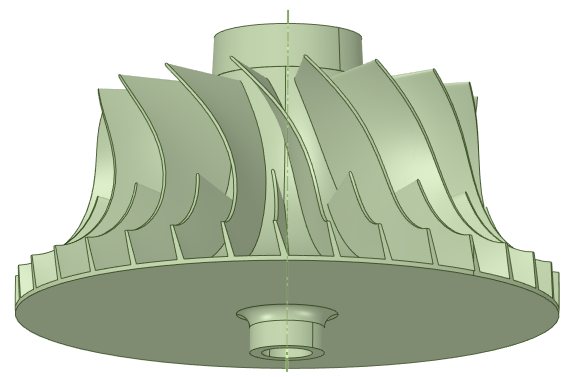a | | 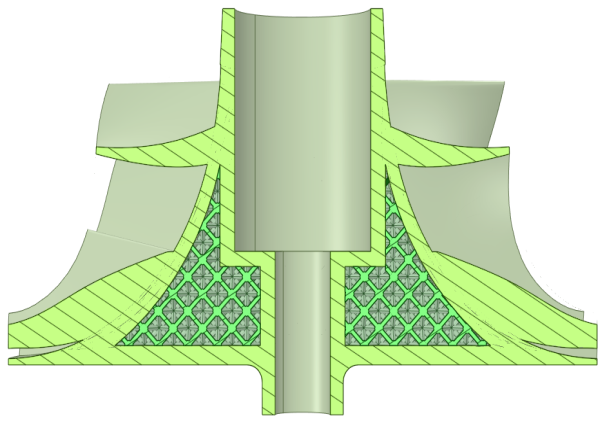b | |
| --- | --- | --- | --- |
| 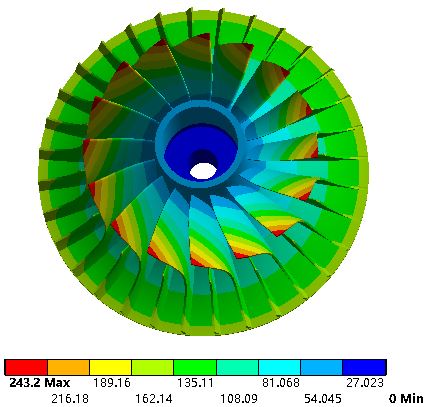c | 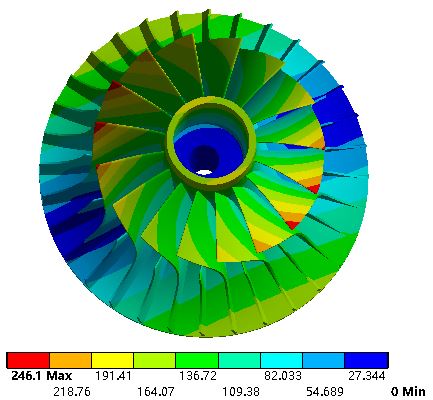d | | 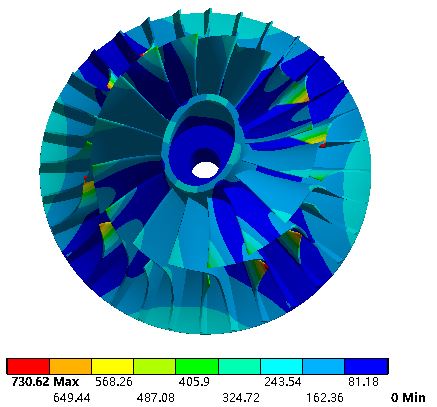e |
| Figure S.1 Solid model of compressor impeller||(a) Solid model of compressor impeller; (b) Sectional view of solid model of compressor impeller; (c) Vibration mode corresponding to 0-nodal-diameter (mm); (d) Vibration mode corresponding to 1-nodal-diameter (mm); (e)Vibration mode corresponding to 2-nodal-diameter (mm). | | | |

| Table S.1 Operation condition of the impellers | | | |
| --- | --- | --- | --- |
| Overall pressure ratio | 4.5 | Inlet pressure | 1.013×10^5^ Pa |
| Rotational | 50000 rpm | Hub diameter | 30mm |
| Vane normal thickness | 8 mm | Shroud diameter | 70mm |
| Vane inlet angle | 60° | Main vanes | 16 |
| Intervanes | 16 | Backsweep angle | 45° |
| Rake angle | 30° |  |  |

| Table S.2 Modal result analysis | |
| --- | --- |
| Nodal-diameter | Frequency (Hz) |
| 0 | 3148 |
| 1 | 3784 |
| 2 | 6186 |

| Table S.3 Main parameters of impeller 3D-printing process | |
| --- | --- |
| Laser power (W) | 275 |
| Laser speed (m/s) | 1.1 |
| Powder thickness (mm) | 0.03 |
| Scanning distance (mm) | 0.12 |
| Base plate temperature (°C) | 200 |
| Initial deflection angle of laser (°) | 15 |
| Layer deflection angle (°) | 67 |

| Table S.4 Density of the powder | |
| --- | --- |
| Temperature (C) | Density (kg m^-3) |
| 20 | 4405 |
| 1227 | 4243 |
| 1777 | 4189 |
| 1877 | 3865 |
| 2127 | 3730 |
| 2500 | 3730 |

| Table S.5 Coefficient of thermal expansion | |
| --- | --- |
| Temperature (C) | Coefficient of Thermal Expansion (C^-1) |
| -233.15 | 6.5E-06 |
| -173.15 | 7.1E-06 |
| 19.85 | 8.9E-06 |
| 126.85 | 9.7E-06 |
| 326.85 | 1.08E-05 |
| 526.85 | 1.14E-05 |
| 626.86 | 1.16E-05 |
| 826.86 | 1.16E-05 |

| Table S.6 Mechanical properties | | | |
| --- | --- | --- | --- |
| Ultimate shear strength | 690 MPa | Ultimate bearing strength | 1350 MPa |

| 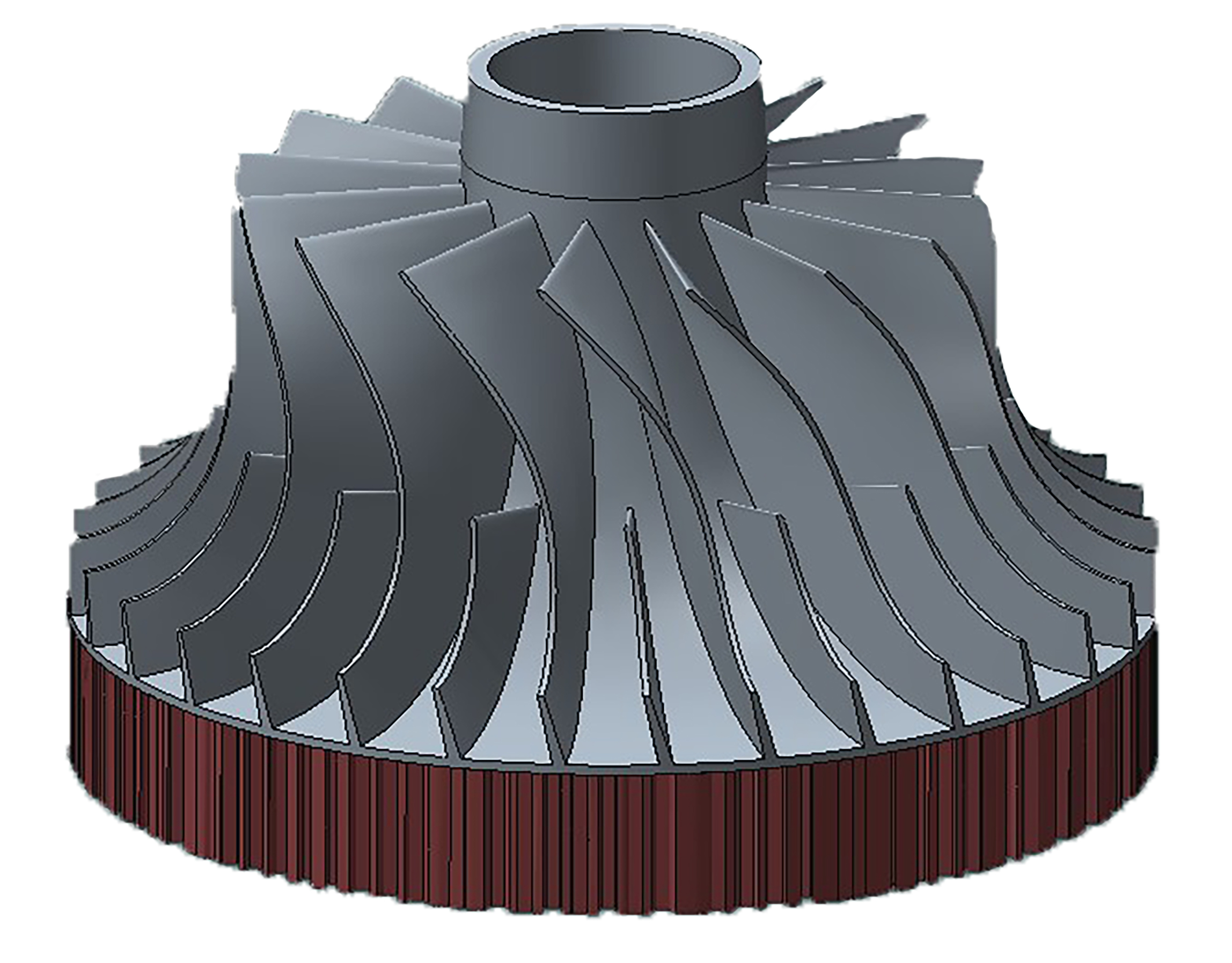 | 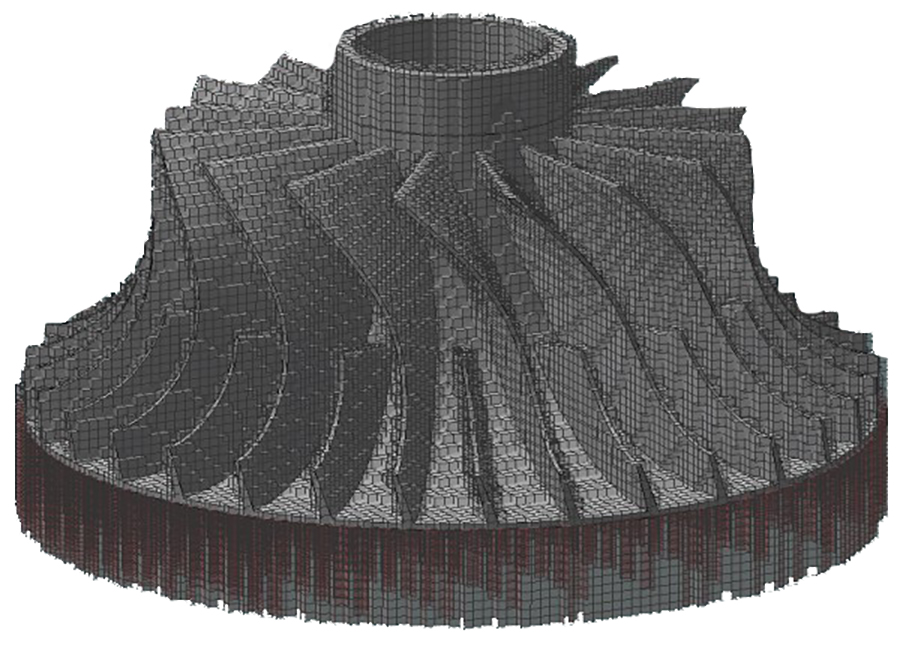 |
| --- | --- |
| Figure S.2 Impeller and support coordination | Figure S.3 Impeller and support coordination |

| 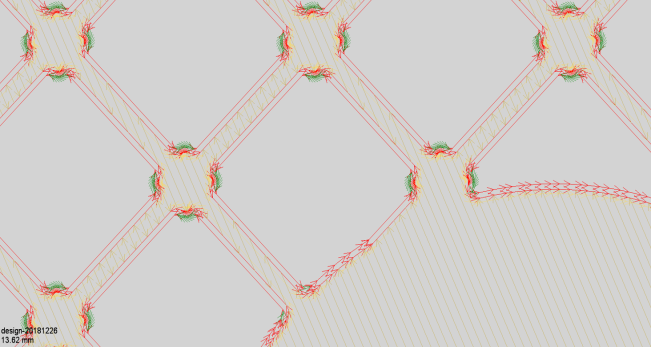a | 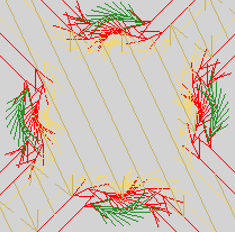b |
| --- | --- |
| 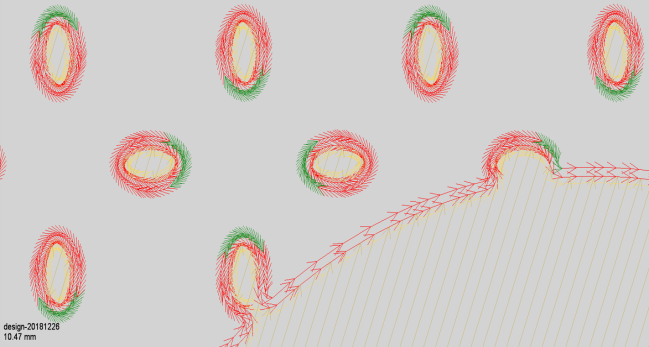c | 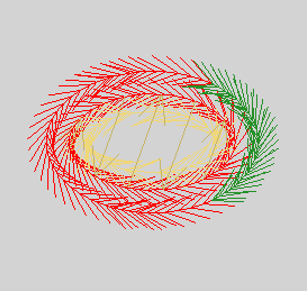d |
| Figure S.4 Some partial laser scanning path|| (a) Scanning path of horizontal section layer of lattice node; (b) Enlargement of a single "Node" path; (c) Scanning path of lattice "Truss" horizontal section layer; (d) Enlargement of single "Truss" section path. The order of scanning is red - orange - yellow - green. | |

| 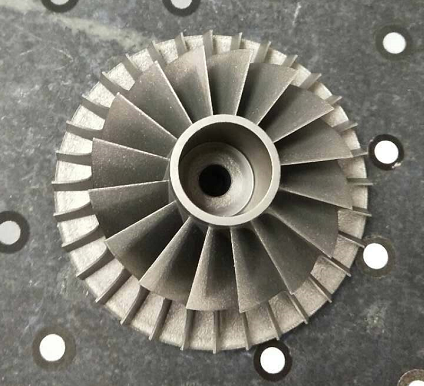a | 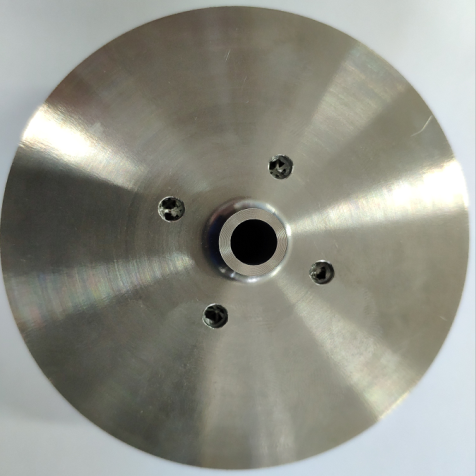b |
| --- | --- |
| Figure S.5 Solid model of impeller|| (a) Impeller top view; (b) Observation of internal lattices through bottom holes of the hub; | |

| 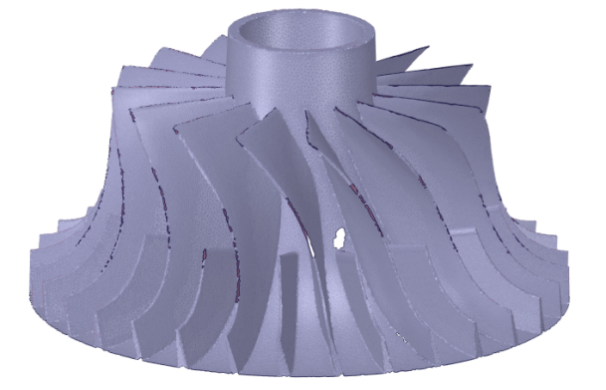a | 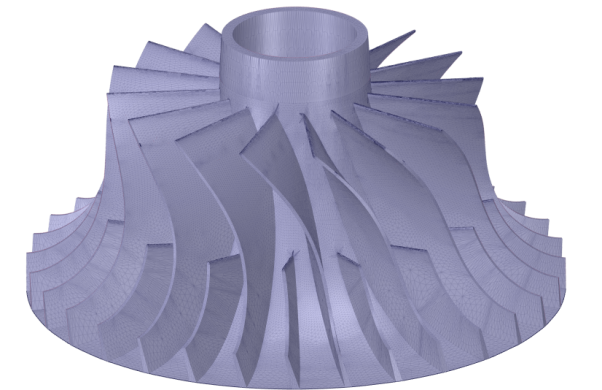b |
| --- | --- |
| Figure S.6 Impeller faceting results|| (a) Scanning geometric files on the surface of the solid model of the impeller. Because of the above-mentioned reasons and the errors in operation, there are a few incomplete surfaces near shroud and smooth surface of the faceted main impeller formed by scanning; (b) Simulated impeller top surface geometry file. Unlike the experimental scanned documents, the derived simulated facet surface is not incomplete. Because a large number of scanned non-fragmented areas can be compared with the simulated export files, a small amount of fragmentation of point cloud geometry files does not influence the comparison of the two results. Because the scanner used in this paper can only obtain the geometric data of the upper surface of the impeller, this section mainly compares the upper surface of the two models. In the future research, X-ray can be used to extract the data of the internal lattice of the impeller and compared with the simulation results. | |

| 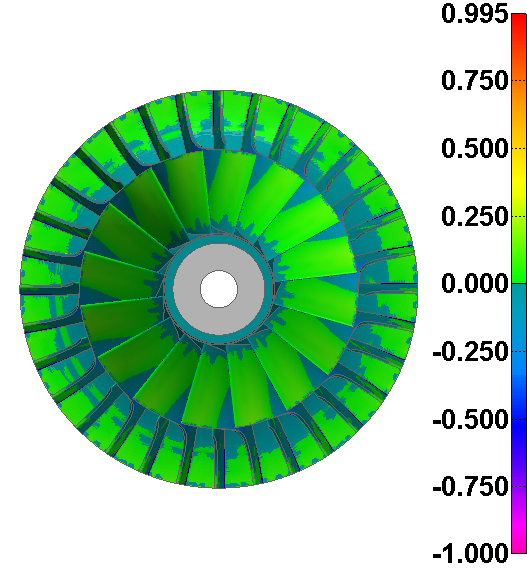a | 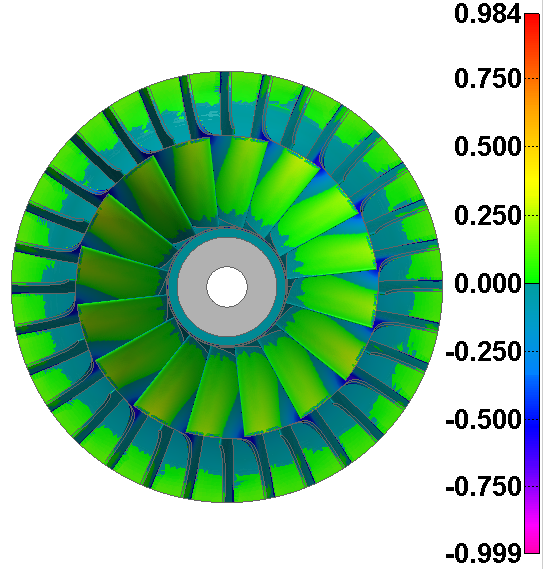b |
| --- | --- |
| 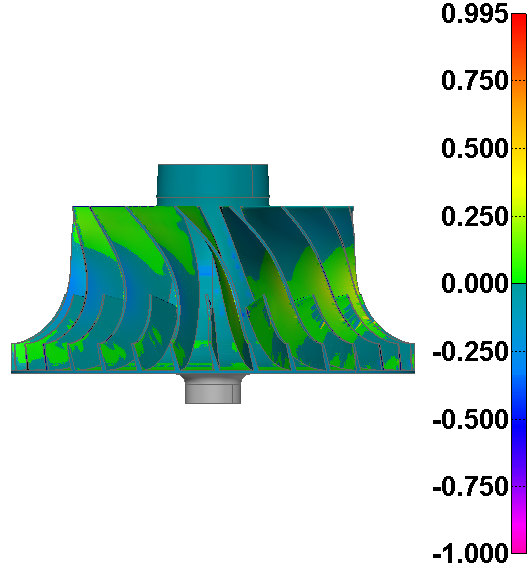c | 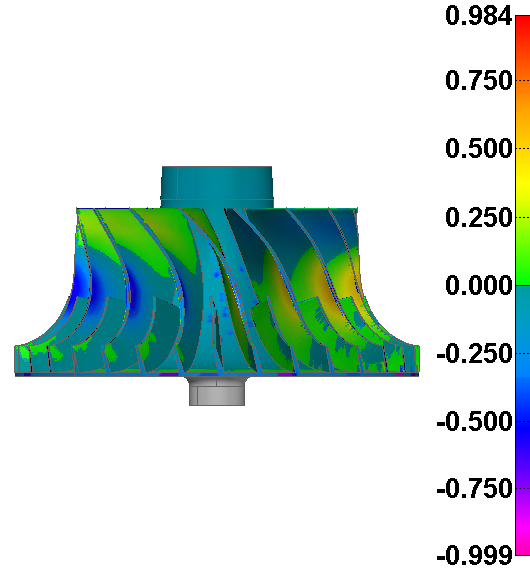d |
| Figure S.7 Comparison of results after surface fitting (mm)|| (a) Impeller solid model-design fitting top view; (b) Impeller simulation-design fitting top view; (c) Impeller solid model-design fitting side view (d) Impeller simulation-design fitting side view. By applying the surface-fitting method, the geometric model of faceted impeller solid obtained by scanning is compared with that of the faceted design model. The fitting results are shown in Figure S.7 (a) and (c). Moreover, the simulation results of faceted impeller geometry model are compared with faceted design model. The fitting results are shown in Figure S.7 (b) and (d). Here, the outer normal direction (positive direction) of the upper surface of the impeller is defined. According to the solid impeller top view in Figure S.7 (a), after printing, there is a small amount of positive displacement on the hub near shroud and the front of the hub of impeller, and a small amount of negative displacement on the leading edge of the blade. This deformation range is generally within 0.2 mm. From the solid impeller side view Figure S.7 (c), it can be seen that after printing, there are some large deformation in the area of the edge near shroud and root of solid model of impeller blade. Owing to the mutual occlusion between the blades, multiple reflections between the emitted laser blades are possible. Therefore, measurement distortion in the larger deformation of the hub is possible. Figures S.7 (b) and (d) show a comparison between the simulation results and the design model. Combined with Figures S.7 (a) and (c), it can be seen that the same distribution trend exists between the simulated deformation and the actual model deformation, and there is a slight difference in the deformation amplitude. It can be concluded that the numerical method presented in this paper is effective in simulating the impeller-printing process. | |

| a | b |
| --- | --- |
| Figure S.8 The maximum residual deformation and stress of the printed piece during the printing process at different laser powers. Here, the number of layers is the number of grid layers, not the number of powder layers. The other parameters of Table S.3 remain unchanged, while the laser power of the printer is changed. In this case, the base plate and support are not yet removed.|| (a) Deformation; (b) Stress. From Figure S.8 (a), it can be seen that in the process of impeller printing, the layer deformation increases with the increase in stacking layers, and there is a non-linear relationship between the maximum layer deformation and the number of layers. Deformation of impeller increases mainly in the first 30 layers (just finishing lattice structure printing); during the first 12 layers of the printing (before the finish the printing of the supporting structure), the deformation of the impeller increases fastest; when the impeller is printed on 31–41 layers, the deformation of the impeller is almost unchanged. In addition, it can be seen from Figure S.8 that at different laser powers, the maximum deformation trends of the impeller during the printing process are similar, but the magnitudes are different. The maximum residual deformation amplitude of impeller increases slightly after the removal of the support and the base plate, due to the different selection of reference points. Figure S.8 (b) depict the maximum stress variation of the impeller during material stacking at different laser powers. From Figure S.8 (b), it can be seen that the maximum stress change in impeller occurs mainly in the first four layers of material stacking during the material stacking process. Moreover, the maximum stress of the first stacking structure increases with the increase in laser power. At nine different laser powers, there are similar trends in the stress variation of the impeller; the maximum stress of impeller increases rapidly and then tends to be stable. | |

| a | b |
| --- | --- |
| Figure S.9 The maximum residual deformation and stress of the printed piece during the printing process at different laser speed. Here, the other parameters of Table S.3 remain unchanged, and the laser speed of the printer is changed. In this case, the base plate and support are not yet removed|| (a) Deformation; (b) Stress. Similar to the case of only changing the laser power, keeping the other parameters of Table 1 unchanged and only changing the laser speed of the printer, the layer deformation increases with the increase in stacking layers in the process of impeller printing. Further, there is a non-linear relationship between the maximum layer deformation and the number of layers. The impeller deformation increases mainly in the first 30 layers (just finishing lattice structure printing); During the first 12 layers of the printing impeller (before finishing the printing process of the supporting structure), the deformation of the impeller increases fastest. When the impeller is printed on 31–41 layers, the deformation of the impeller is almost unchanged. In addition, at different laser speeds, the maximum deformation trends of impeller in the printing process are similar, but the amplitude is different. The overall trend of residual deformation amplitude of compressor impeller decreases with the increase in laser speed. Figure S.9 (b) depict the maximum stress variation of the impeller during material stacking at different laser speeds. From Figure S.9 (b), it can be seen that in the process of material stacking, the greatest change in impeller stress occurs mainly in the process of material stacking in the first four layers. Moreover, with the increase in laser speed, the maximum stress of the first stack structure increases. At nine different laser powers, the stress variation of the impeller has a similar trend; the maximum stress of the impeller increases rapidly and then tends to be stable. | |

| a | b |
| --- | --- |
| Figure S.10 The maximum residual deformation and stress of the printed piece during the printing process at different laser widths. Here, the laser width of the printer is changed and the other parameters of Table S.3 remain unchanged. In this case, the base plate and support have not yet been removed|| (a) Deformation; (b) Stress. From Figure S.10, it can be seen that the layer deformation increases with the number of stacked layers, and there is a similar non-linear relationship between the maximum layer deformation and the number of layers, as in the previous relationship. The stress change of the impeller has a similar trend; the maximum stress of impeller increases rapidly and then tends to be stable. | |

| a | b |
| --- | --- |
| Figure S.11 The maximum residual deformation and stress of the printed piece during the printing process at different powder thickness. Here, the powder thickness of the printer is changed and the other parameters of Table S.3 remain unchanged. In this case, the base plate and support have not yet been removed|| (a) Deformation; (b) Stress. Compared with the printing conditions with different laser parameters, the variation in the thickness of the powder coating in a small range has little effect on the amplitude of the residual deformation and stress in the time domain during the printing process. In this case, the residual deformation amplitude and residual stress amplitude of the printed piece in the printing process still have a similar relationship with the corresponding amplitude in the printing operation with different laser parameters. | |

Table S.7 Printing parameters at different printing conditions

| Work case | Laser power (W) | Laser speed (m/s) | Powder layer thickness (μm) | Laser width (mm) |
| --- | --- | --- | --- | --- |
| 1 | 275 | 1.1 | 30 | 0.12 |
| 2 | 300 | 1.1 | 30 | 0.12 |
| 3 | 275 | 1.1 | 30 | 0.15 |
| 4 | 275 | 1.1 | 50 | 0.12 |
| 5 | 275 | 1.5 | 30 | 0.12 |

| 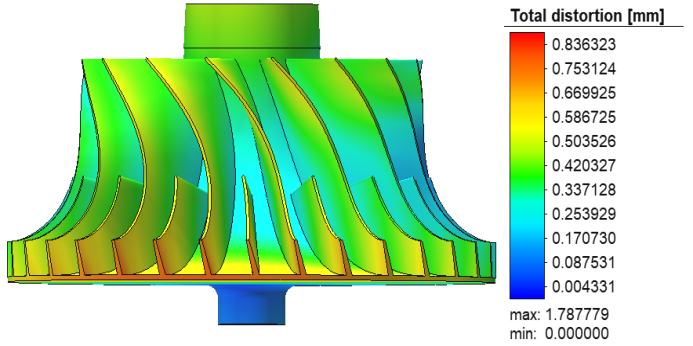a | 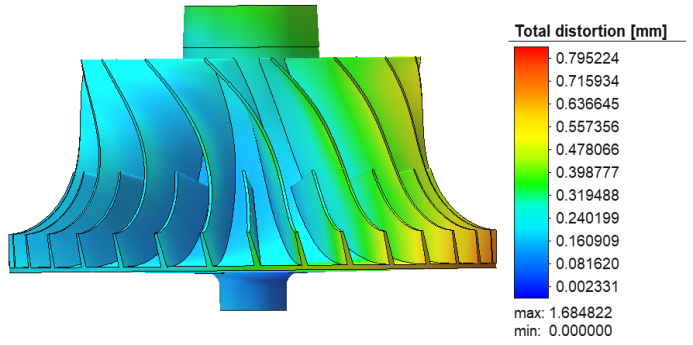b |
| --- | --- |
| 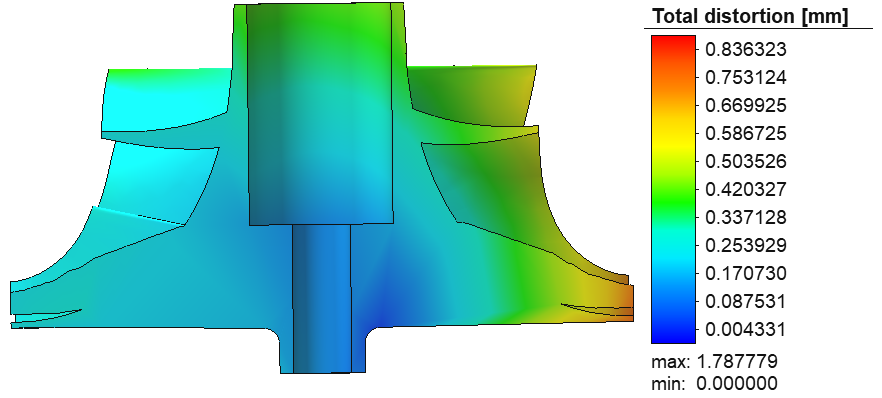c | 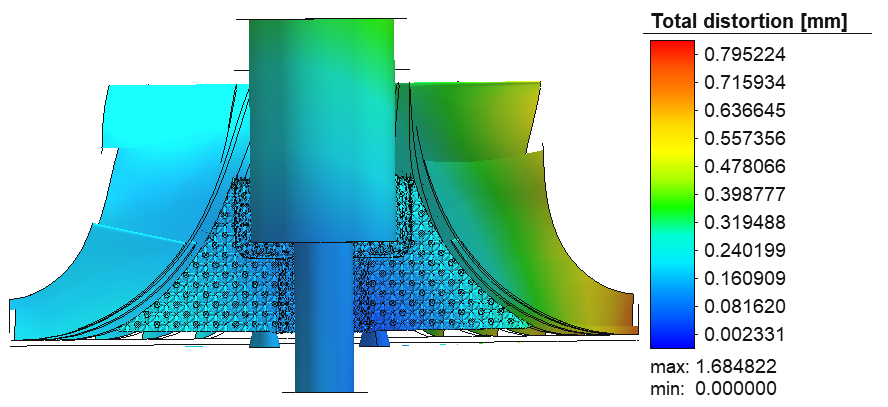d |
| 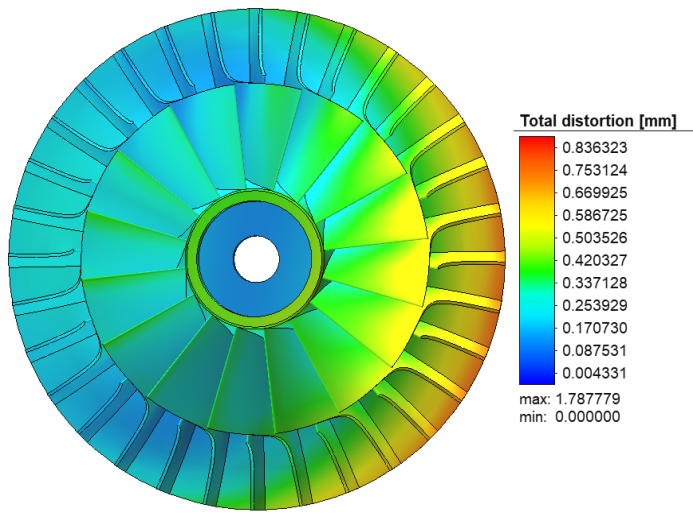e | 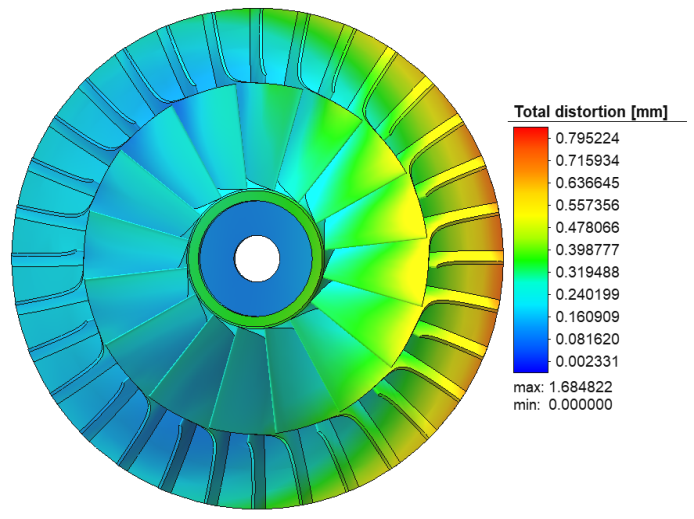f |
| 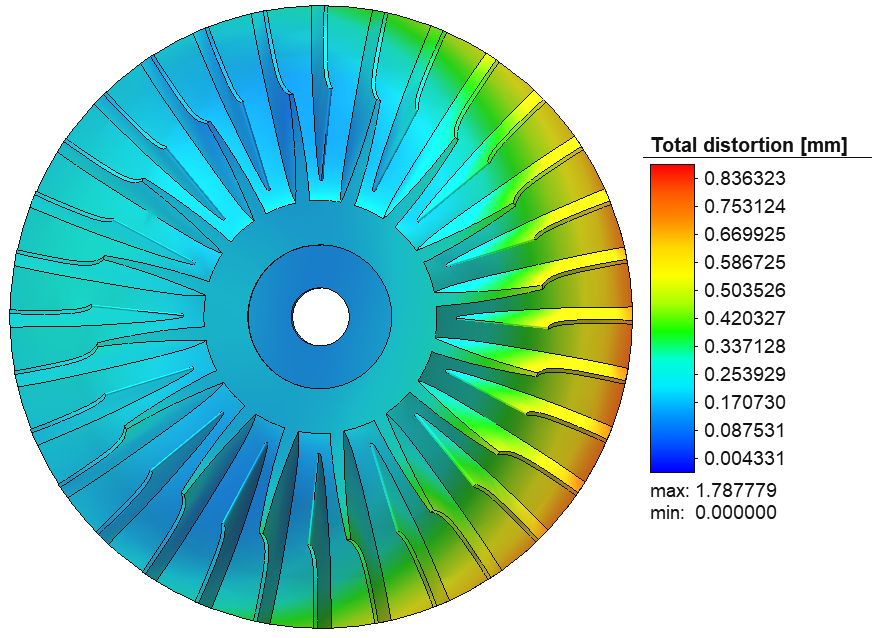g | 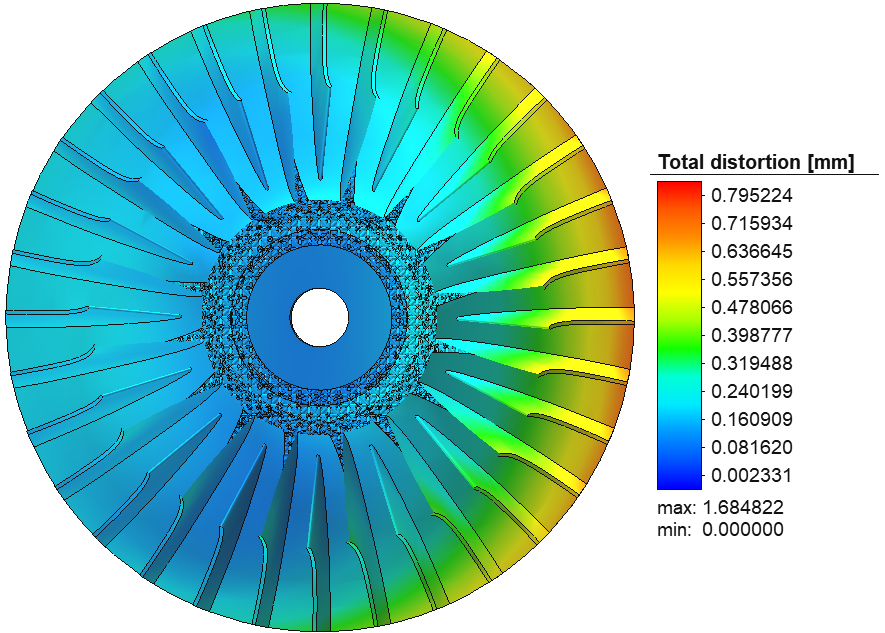h |
| Figure S.12 Residual deformation distribution of solid impeller and lattice impeller after removal of base plate and support at printing work case 1||(a) Side view of residual deformation of solid impeller; (b) Side view of residual deformation of lattice impeller; (c) Side section view of residual deformation of solid impeller; (d) Side section view of residual deformation of lattice impeller; (e) Top view of residual deformation of solid impeller; (f) Top view of residual deformation of lattice impeller; (g) Top view of residual deformation of solid impeller (with section); (h) Top view of residual deformation of lattice impeller (with section). | |

| 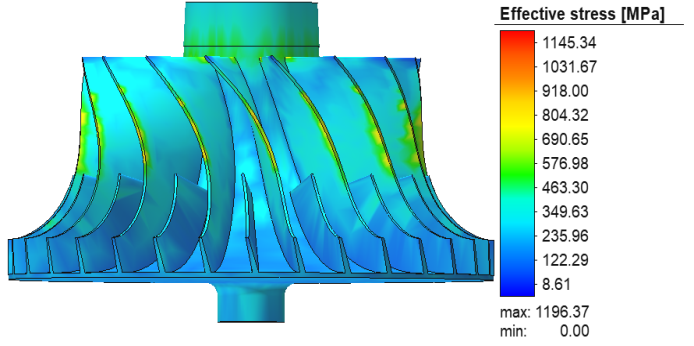a | 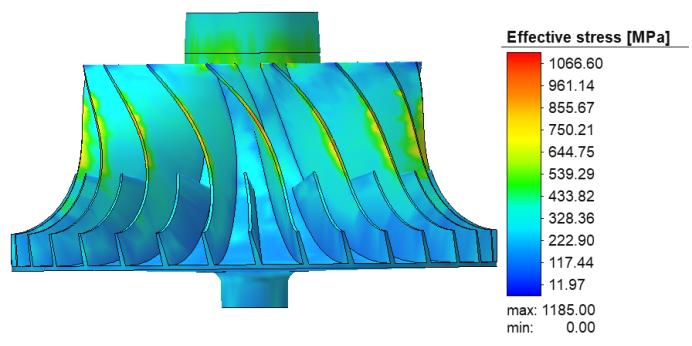b |
| --- | --- |
| 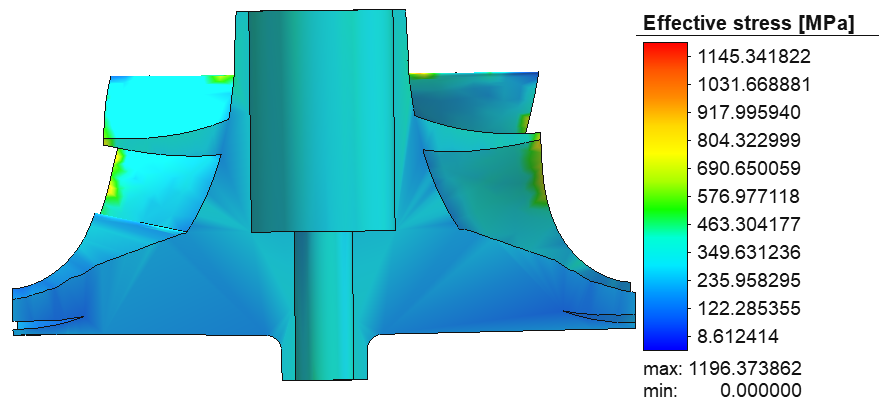c | 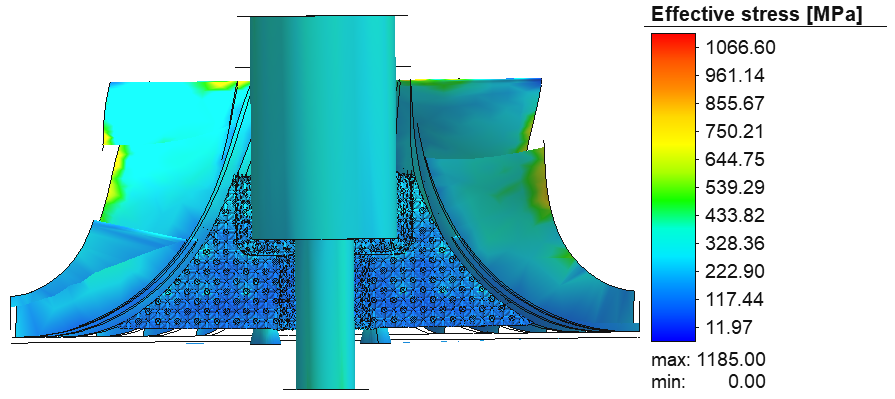d |
| 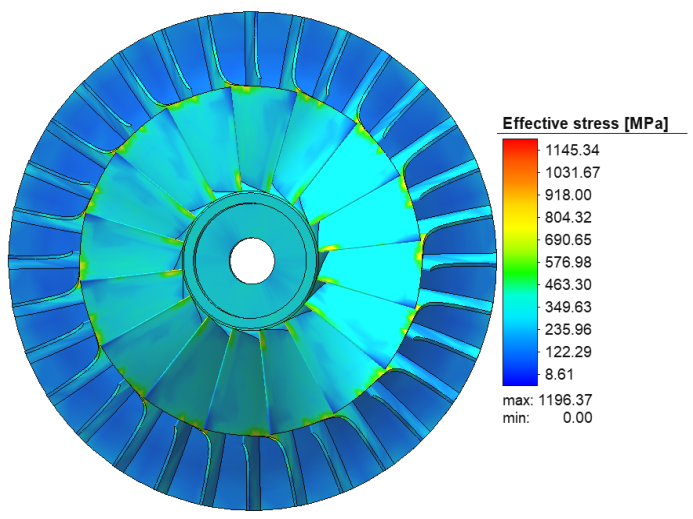e | 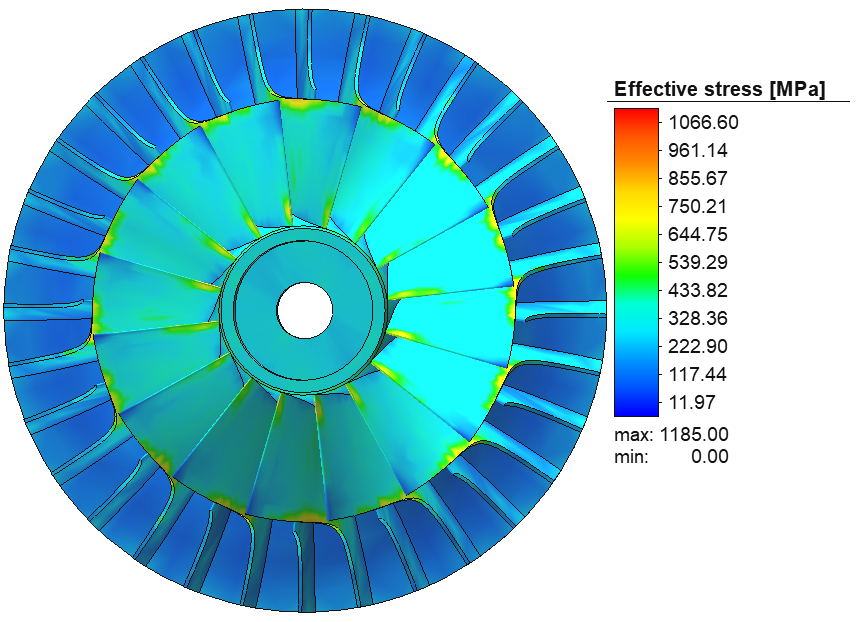f |
| 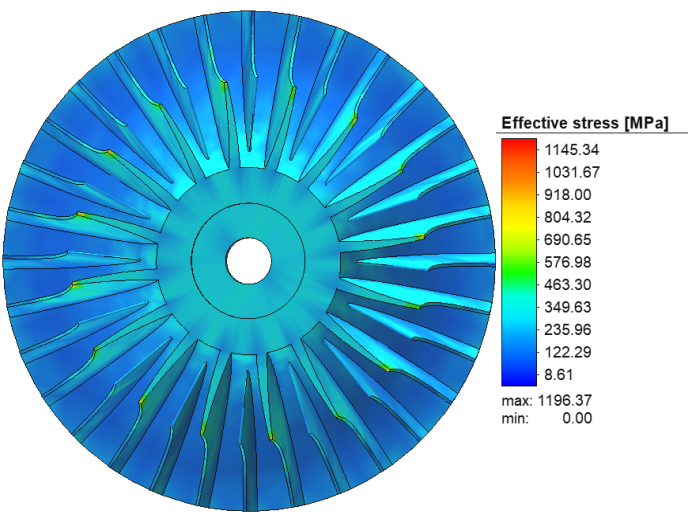g | 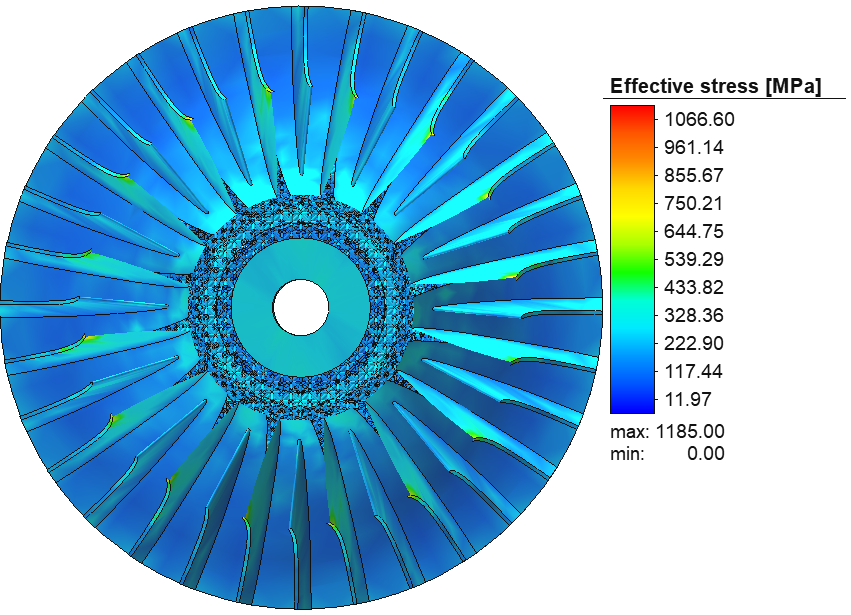h |
| Figure S.13 Residual deformation stress of solid impeller and lattice impeller after removal of base plate and support at printing work case 1|| (a) Side view of residual stress of solid impeller; (b) Side view of residual stress of lattice impeller; (c) Side section view of residual stress of solid impeller; (d) Side section view of residual stress of lattice impeller; (e) Top view of residual stress of solid impeller; (f) Top view of residual stress of lattice impeller; (g) Top view of residual stress of solid impeller (with section); (h) Top view of residual stress of lattice impeller (with section). | |
